# Mobility of the human foot’s medial arch helps enable upright bipedal locomotion

**DOI:** 10.3389/fbioe.2023.1155439

**Published:** 2023-05-30

**Authors:** Lauren Welte, Nicholas B. Holowka, Luke A. Kelly, Anton Arndt, Michael J. Rainbow

**Affiliations:** ^1^ Department of Mechanical and Materials Engineering, Queen’s University, Kingston, ON, Canada; ^2^ Department of Anthropology, University at Buffalo, Buffalo, NY, United States; ^3^ School of Human Movement and Nutrition Sciences, University of Queensland, Brisbane, QLD, Australia; ^4^ The Swedish School of Sport and Health Sciences (GIH), Stockholm, Sweden; ^5^ Karolinska Institute, Stockholm, Sweden

**Keywords:** foot lever, arch spring, biplanar videoradiography, walking, running, longitudinal arch, foot biomechanics

## Abstract

Developing the ability to habitually walk and run upright on two feet is one of the most significant transformations to have occurred in human evolution. Many musculoskeletal adaptations enabled bipedal locomotion, including dramatic structural changes to the foot and, in particular, the evolution of an elevated medial arch. The foot’s arched structure has previously been assumed to play a central role in directly propelling the center of mass forward and upward through leverage about the toes and a spring-like energy recoil. However, it is unclear whether or how the plantarflexion mobility and height of the medial arch support its propulsive lever function. We use high-speed biplanar x-ray measurements of foot bone motion on seven participants while walking and running and compare their motion to a subject-specific model without arch recoil. We show that regardless of intraspecific differences in medial arch height, arch recoil enables a longer contact time and favorable propulsive conditions at the ankle for walking upright on an extended leg. The generally overlooked navicular-medial cuneiform joint is primarily responsible for arch recoil in human arches. The mechanism through which arch recoil enables an upright ankle posture may have helped drive the evolution of the longitudinal arch after our last common ancestor with chimpanzees, who lack arch plantarflexion mobility during push-off. Future morphological investigations of the navicular-medial cuneiform joint will likely provide new interpretations of the fossil record. Our work further suggests that enabling medial arch recoil in footwear and surgical interventions may be critical for maintaining the ankle’s natural propulsive ability.

## 1 Introduction

The foot experienced strong selective pressures during human evolution. Features unique to the human foot, such as a pronounced medial arch, have been proposed to play a key role in the evolution of habitual bipedalism (Elftman and Manter, 1935b). The presence of a high medial arch in fossil hominins has been argued to represent an adaptation for both the foot’s levering ability in push-off (Elftman and Manter, 1935a; Susman, 1983; Sarrafian, 1987) and its mobility-enabled spring-like function (Hicks, 1955; Holowka and Lieberman, 2018; McNutt et al., 2018). Both functions are thought to assist with propulsion of the body in late stance: in theory, the curved longitudinal arch provides a nearly rigid lever for push-off, similar to ancient architecture (Morton, 1924), while the mobility of various foot joints enables the spring-like recoil of the arch-spanning tissues (Ker et al., 1987; Kelly et al., 2014; Stearne et al., 2016). However, it remains unclear how arch recoil interacts with its lever function to enable locomotion.

The arch of the human foot recoils (plantarflexes) in propulsion, in contrast to the feet of our closest living relatives, chimpanzees, whose midfoot joints initially dorsiflex after heel lift (Holowka et al., 2017a). In fact, all non-human primates where midfoot motion has been observed experience a similar midfoot “break” (Elftman and Manter, 1935a; D’Août et al., 2002; Vereecke et al., 2003; DeSilva, 2009; Griffin et al., 2010). The hindfoot lifts relative to the ground-contacting metatarsals, forming a “reverse-arch” where the midfoot is below the plane connecting the heel and the toes (Bennett et al., 1989). The fulcrum of the foot-lever becomes the midfoot instead of the metatarsophalangeal joints (Griffin et al., 2010), shortening the foot-lever and reducing the mechanical advantage. This midfoot mobility may provide advantages for climbing (Elftman and Manter, 1935a; Bojsen-Møller, 1979; Holowka et al., 2017b) but is thought to reduce the efficiency of push-off when walking bipedally. In contrast, humans’ robust plantar fascia and plantar ligaments, as well as the elevated arch structure, contribute to the stability of the longitudinal arches (Bojsen-Møller, 1979; DeSilva, 2009; Sichting et al., 2020). At heel lift, they keep the midfoot above the plane connecting the heel and the toes. Some humans experience a slight midfoot break, but not to the extent of other primates (Greiner and Ball, 2014; DeSilva et al., 2015). As a result, the fulcrum of the human foot-lever is primarily the metatarsophalangeal joints (Hicks, 1954; Griffin et al., 2010), enabling humans to take advantage of additional leverage relative to primates with a midfoot break. While the fulcrum of the non-human primate foot lever can be influenced by midfoot mobility, it remains unclear how human arch plantarflexion influences its function as a lever. Understanding this mechanism may further elucidate our evolutionary divergence from other primates.

**FIGURE 1:**
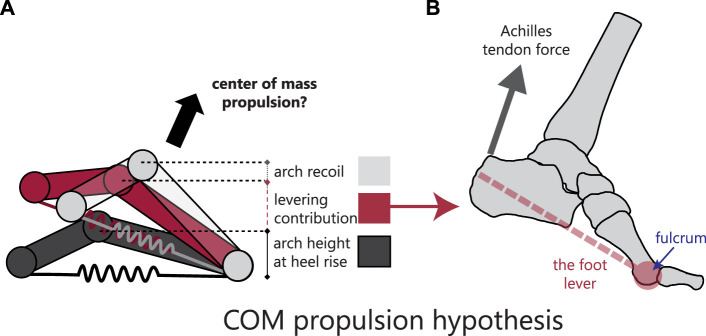
**(A)** The center of mass (COM) propulsion hypothesis suggests that both arch recoil and the foot’s levering ability can raise the COM. **(B)** The foot levers around the fulcrum metatarsophalangeal joint.

During late stance of walking and running, the arch of the foot recoils substantially (Kelly et al., 2014). When overall arch motion is restricted in running, metabolic cost increases, suggesting that arch recoil may benefit whole body locomotion (Stearne et al., 2016). However, the mechanism of this arch recoil benefit to locomotion is less clear. One hypothesis is that recoil of the arch-spanning tissues makes up mechanical work that would otherwise be produced at a metabolic cost by muscles in parallel (Stearne et al., 2016). Alternatively, the arch could contribute to propulsion of the center of mass (COM). Running is consistently modelled as a spring-mass system, where the leg-spring extends in propulsion, moving the COM forwards and upwards (Ker et al., 1987; Blickhan, 1989; Kelly et al., 2014; Stearne et al., 2016). In theory, the arch can contribute to this leg-spring extension by lifting the apex of the foot, by approximately 10–15 mm (Stearne et al., 2016) (Figure 1A). However, arch recoil is small in comparison to COM excursion, which is closer to 80 mm (Lee and Farley, 1998; McKinon et al., 2004). While walking is modelled less simply than the leg-spring model, researchers have had success modelling COM trajectories using a spring-loaded inverted pendulum model (Geyer et al., 2006). This model can be improved by adding a compliant foot-ankle (Lim and Park, 2018), suggesting that arch recoil may also contribute to COM propulsion in walking. However, the hypothesis that *in vivo* arch recoil contributes to center of mass propulsion has not been tested in either walking or running, and the mechanisms through which *in vivo* arch recoil benefits locomotion remain unclear.

An alternative hypothesis to explain the role of late stance foot arch recoil is that its shape and plantarflexion mobility are critical for allowing the ankle and proximal segments to achieve postures required for efficient locomotion (Lim and Park, 2018). Distal body segment movement can influence the proximal segment’s global motion, suggesting the arch’s substantial motion could affect ankle function. This idea is inspired by previous work showing that restricting ankle dorsiflexion prior to heel-off leads to additional knee extension in propulsion (Ota et al., 2014). Thus, as part of the leg chain, when the medial column of the foot behaves as a dynamic lever that pushes off the ground, it lifts the ankle (Figure 1B). However, as a dynamic lever, arch recoil can change the length and posture of the lever, affecting ankle global position and orientation (Smith et al., 2022). When arch recoil is surgically restricted, patients have significantly reduced ankle power generation, further suggesting that arch recoil influences ankle kinetics (Beischer et al., 1999). Ankle posture during gait may also be affected by static arch type, i.e., a low or high arch, where a high arch achieves push-off postures faster than a low arch, as the high arch would have less distance to travel. If the arch is not high enough or does not sufficiently recoil, the ankle may display reduced range of motion, placing the tibia in a sub-optimal posture during gait.

Here, using *in vivo* measurements of individual foot bone motion derived from biplanar videoradiography, we test the hypotheses that the arch recoils to propel the COM and that arch recoil influences the posture of the ankle. First, we compare the timing of COM propulsion and arch recoil. We predict that the timing of COM propulsion and arch recoil will be synchronized in walking and running, consistent with an extending leg-spring. Second, we developed a model that mathematically restricts arch recoil but maintains levering about the metatarsophalangeal joint (MTPJ). We expect that the talus, as the apex of the arch, will be higher at push-off in an arch that recoils, compared to an arch that recoils less, or not at all (Figure 1A). We place these findings in evolutionary context with data of chimpanzees walking bipedally, who have a midfoot that plantarflexes significantly less than in humans at heel-lift. Additionally, we predict that kinematic parameters that affect ankle propulsion (such as ankle plantarflexion and ground contact time) will be affected by arch plantarflexion mobility. Third, to examine whether arch shape (static arch height) and dynamic motion (arch recoil) influence the global position of the ankle during the propulsive phase of gait, we examined participants with a range of arch plantarflexion mobility and foot types, and identify which joints within the foot contribute most to its recoil.

## 2 Materials and methods

### 2.1 Biplanar videoradiography dataset

Seven young, physically active subjects (4F, 3M, mean ± 1SD, 23.3 ± 3.0 years, 1.72 ± 0.08 m, 69.6 ± 7.6 kg, short IPAQ (Craig et al., 2003) moderate & vigorous physical activity: 477 ± 325 min/week) were selected from a 20 participant pool to span the observed range of static arch heights. The selected participants walked and ran overground at a self-selected speed (walking (W) 1.6 ± 0.1 m/s, running (R) 3.0 ± 0.4 m/s) in flexible, thin-soled minimal shoes (7.5 mm sole, 0 mm heel-toe drop, Xero Prio Shoes, Broomfield, CO, United States) while biplanar videoradiography captured their foot bone motion (Skeletal Observation Lab, Queen’s University, Kingston, ON, Canada). The experimental protocol was approved by Queen’s University Health Sciences and Affiliated Teaching Hospitals Research Ethics Board. All participants gave informed consent prior to participation in the data collection.

Three-dimensional positions and orientations of individual foot and ankle bones (tibia, calcaneus, talus, navicular, medial cuneiform, first metatarsal and first proximal phalanx) were measured using X-ray Reconstruction of Moving Morphology (Brainerd et al., 2010; Knörlein et al., 2016). This technology combines high-speed biplanar videoradiography ((W) 125 Hz, (R) 250 Hz) with bone models derived from a computed tomography (CT) scan to visualize rapid skeletal movement *in vivo*.

Three high-speed x-ray trials were collected for each participant (71 kV, 125 mA, shutter speed (W) 1250 µs (R) 1000 µs, resolution 2048 × 2048 pixels) as their right foot landed and pushed off in the x-ray capture volume. One trial was selected for analysis for x-ray image quality and appropriate participant foot placement. The biplanar videoradiography collection pipeline for the foot bones has been described previously (Kessler et al., 2019). Briefly, the high-speed cameras were calibrated using a custom calibration object, and the images were undistorted using open-source x-ray processing software (Knörlein et al., 2016) (XMALab, Providence, RI, United States).

A CT scan was taken of each participant’s right foot while supine, with a maximally plantarflexed ankle for improved in-plane resolution (Revolution HD; General Electric Medical Systems, Chicago, IL, United States; resolution: 0.317 mm × 0.317 mm x 0.625 mm). All bones (tibia, calcaneus, talus, navicular, medial cuneiform, first metatarsal, first proximal phalanx and first distal phalanx) were segmented (Mimics, Materialise, Leuven, Belgium). Tessellated meshes depicted the bone surfaces and were used to establish inertial coordinate systems. The coordinate systems’ origin was located at the centroid of each bone, and the three axes aligned with the principal directions of the moment of inertia tensor (Eberly et al., 1991). The axes were re-labelled such that the *x*-axis was lateral, with positive angles about this axis indicating dorsiflexion. Specialized coordinate systems with a cylinder fitted to the talar and tibia domes were used to measure ankle dorsi/plantarflexion.

Partial volumes generated from the bone masks formed digitally reconstructed radiographs (Miranda et al., 2011). Custom software (Autoscoper, Brown University, Providence, RI, United States) semi-manually measured the orientation and translation of the bones of interest using the digitally reconstructed radiographs and undistorted x-ray images. The digitally reconstructed radiographs were manually aligned with the two x-ray views, and a particle swarm algorithm optimized the normalized cross-correlation values (Akhbari et al., 2021). A 3D visualization of the bone positions ensured that no collisions occurred between adjacent bones (Welte et al., 2022a).

### 2.2 Angles

Dorsiflexion (+)/plantarflexion (−) is measured as the Tait-Bryan angle of the distal bone relative to the proximal bone using a YZX sequence to prioritize *x*-axis dorsi/plantarflexion. MTPJ dorsiflexion measures the first proximal phalanx’s motion relative to the first metatarsal. Arch plantarflexion and dorsiflexion refer to the first metatarsal’s sagittal motion relative to the calcaneus and are referred to as arch angle. Arch angle is described as flattening (dorsiflexion) in early- and mid-stance, and as recoil (plantarflexion) in propulsion. Ankle dorsi/plantarflexion measures the talus’s motion relative to the tibia. Range of motion for ankle plantarflexion and arch recoil is measured between peak arch flattening and peak MTPJ dorsiflexion.

To measure the contributions of the medial column to propulsion, we measured the plantarflexion of each arch bone-pair (talonavicular, cuneonavicular, cuneometatarsal) using the orientation of the inertial coordinate system of the first metatarsal, aligned at peak arch flattening (Supplementary Figure S1). The convention for this analysis was to report plantarflexion as positive by switching the sign of the Tait-Bryan angle. The range of motion for each joint was again measured between peak arch flattening and peak MTPJ dorsiflexion. A Friedman test examined the differences in range of motion at each joint with a Bonferroni correction for multiple comparisons between the joints.

Tibial lean was measured as the global orientation of the tibia relative to the global axes. The X angle of a Tait-Bryan YZX sequence of the tibial inertial coordinate system relative to the global axis measured the tibial lean. The tibial anatomical coordinate system was defined specifically for this measurement, with the *z*-axis aligned with a cylinder fitted to the long axis of the tibial shaft. The *x*-axis is directed laterally (approximately originating at the medial malleolus), and the *y*-axis is the anteriorly directed mutual perpendicular to the *x*- and *z*-axes.

### 2.3 Center of mass calculation

Eight optical motion capture cameras (Qualisys, Sweden) captured the positions of retroreflective markers affixed to the body (Kanko et al., 2021). The center of mass was calculated using rigid segment definitions in Visual3D (C-Motion, Kingston, ON, Canada) for the pelvis, torso, and the bilateral shanks, thighs, upper arms, and forearms. One participant’s walking data was omitted due to poor quality marker data.

### 2.4 Arch height

Arch height was measured in a static seated position. The participants were instructed to place their right barefoot in front of their left foot in the capture volume and to distribute their weight evenly between their legs. The position and orientation of the arch bones and first proximal phalanx were measured using the previously described methods for processing biplanar x-ray data.

A slightly modified arch height index (AHI) contextualized the range of foot types (Butler et al., 2008). AHI is typically measured with a specialized device and is the quotient of the arch height at 50% of the total foot length and the truncated foot length. Here, the medial arch was oriented by the principal component axes of the arch bone vertices. The first principal component represented the anterior direction of the foot and the second principal component represented the height of the arch. The truncated foot length measured the distance from the most posterior point of the heel to the anterior tip of the first metatarsal. As we could not measure the pose of the longest distal phalanx, we fixed the first distal phalanx with the first proximal phalanx. Foot length was measured from the previously calculated posterior heel point to the tip of the first distal phalanx. The highest vertex of the arch bones within 1 mm of the 50% length of the foot was selected as the dorsal arch height. The dorsal arch height was then divided by the truncated foot length to measure AHI.

Arch angle was also measured in the static seated position. AHI and arch angle were linearly correlated (*p* < 0.01, *R* ^2^ = 0.87); thus, we selected arch static dorsiflexion angle for our analyses as it was more similar to our dynamic measures.

### 2.5 Rigid foot model

We tested the contributions of a rigid arch to propulsion by mathematically locking the arch bones with the first metatarsal at the beginning of arch recoil (see Supplementary Material S1), which is the flattest arch position during stance. The arch posture (i.e., fully flat or fully recoiled) did not change our outcomes. The arch bones, with no relative motion between them, were driven with the motion of the first metatarsal through propulsion. The end of propulsion was defined to be the maximum MTPJ dorsiflexion angle. Relative tibiotalar motion remained consistent between rigid and moving arch propulsion. Comparisons were made between the modelled rigid arch and the measured naturally recoiling arch for each participant. All modelling and optimization described in this section was conducted in MATLAB R2020b (Mathworks, Natick, MA, United States).

To test the contributions of the foot’s levering motion (rigid) and arch recoil (moving) to ankle posture, we measured the location of the talar centroid for the rigid and recoiling arch. The talar centroid was projected into the inertial coordinate system of the first proximal phalanx to standardize the direction of take-off among participants. We measured the height and forward progression of the talar centroid in three analyses: first, at the end of propulsion, with the same contact time and therefore levering motion between rigid and moving arches; second, when the rigid arch’s tibia aligned with the moving arch’s tibia at push-off; and third, with both levering motion and push-off tibia position maintained. In the second analysis, tibia alignment was measured as the maximum value of the dot product of the vector aligned with the tibial shaft in each condition. In the third analysis, the tibia was rotated about its helical axis by the angle between the global positions of the rigid and moving-arch tibiae. The translation of the newly rotated tibia was optimized such that there were no bone collisions while minimizing the mean distance between the tibia and talus.

A two-tailed Wilcoxon signed-ranks test measured the difference in talar height between the rigid and moving arches for walking and running as Shapiro-Wilks normality tests indicated that the distributions were not normal. Significance was set at α = 0.05. Effect sizes are reported as the difference between the proportions of favorable and unfavorable outcomes, with 0 indicating no effect and 1 indicating all pairs behaved the same way (Kerby, 2014). All reported values are mean ± one standard deviation unless otherwise indicated.

### 2.6 Multiple regression model

The influence of arch plantarflexion mobility and arch height on ankle kinematic measurements was analyzed using two multiple regression models. The predictor variables were arch recoil range of motion, static arch angle, and a categorical variable indicating whether the trial was a walk or a run. The response variables were the magnitude of talar displacement in the anterior and superior directions (from peak arch flattening to peak MTPJ dorsiflexion) and ankle plantarflexion range of motion. Assumptions of variable collinearity and homoscedasticity, as well as independence and normality of residual values, were met. Significance was set at α = 0.05. Statistical analysis was completed in MATLAB R2020b (Mathworks, Natick, MA, United States), using the fitlm function.

### 2.7 Chimpanzee dataset

Chimpanzee data were collected previously from three subadult male chimpanzee subjects (age: 5.5 ± 0.2 yrs; 26.5 ± 6.7 kg) (O’Neill et al., 2015). Chimpanzees were housed at an Association for Assessment and Accreditation of Animal Laboratory Care International-approved facility, and all experimental protocols were approved by Stony Brook University’s Institutional Animal Care and Use Committee. Chimpanzees were previously trained to walk bipedally using positive reinforcement. During data collection, chimpanzees were encouraged to walk bipedally by an animal trainer who used food and juice rewards. Four high-speed video cameras recording at 150 Hz were used to capture motion as the subject walked on a flat 11-meter runway at self-selected speeds. We analyzed a total of 13 bipedal steps (Subject A: 5 steps; Subject B: 1 step; Subject C: 7 steps), and subjects walked at an average speed of 1.13 ± 0.11 m/s (0.35 ± 0.09 Froude). Tibial lean was calculated as the angle between the global vertical axis and the vector connecting markers that were applied to the lateral malleolus and the fibular head.

## 3 Results

### 3.1 Center of mass propulsion

#### 3.1.1 Timing of COM propulsion

**FIGURE 2:**
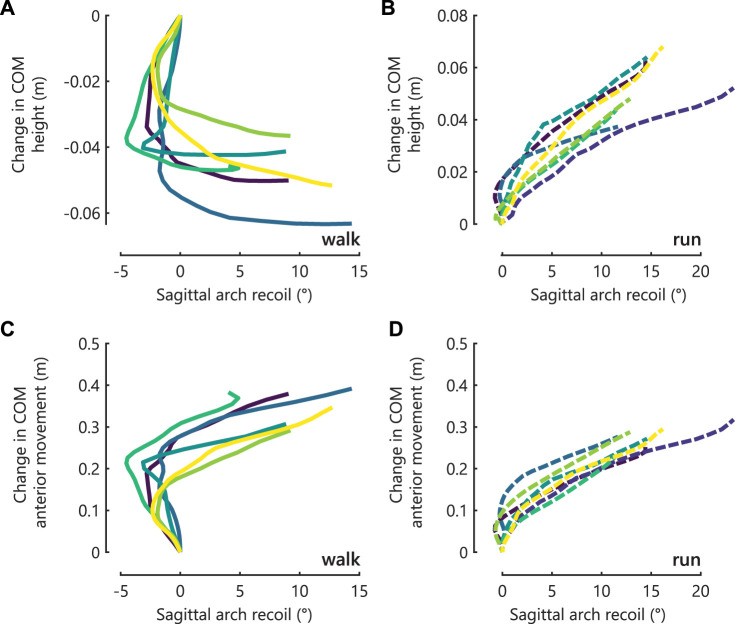
The COM position as related temporally to sagittal arch recoil from the beginning of the propulsive ground reaction force until maximum metatarsophalangeal joint dorsiflexion. The height of the COM with respect to its height at the beginning of propulsion is shown in **(A)** walking (solid lines) and **(B)** running (dashed lines). The anterior movement of the COM is shown in **(C)** walking and **(D)** running. Each line color represents a participant, which is consistent across all panels.

During walking, the superior movement of the COM was temporally offset with arch recoil (Figure 2). The COM moved inferiorly while the arch maintained the same height, and then rose minimally while the arch recoiled (Figure 2A). Approximately halfway through the propulsion in the anterior direction, the arch recoiled synchronously with the anterior movement of the COM (Figure 2C).

During running, arch recoil increased with COM movement in both the anterior and superior directions (Figures 2B, D).

### 3.2 Talus posture

#### 3.2.1 Model

**FIGURE 3:**
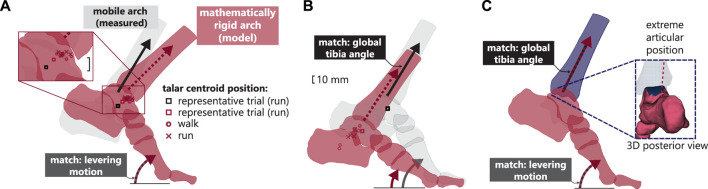
The contributions of arch recoil (measured using high speed x-ray, light gray) with respect to the modelled rigid arch (arch recoil mathematically removed, red) for a representative running trial. The position of the talar centroid of the rigid arch for each participant (o: walk, x: run) is shown with respect to the measured talar centroid position when **(A)** the levering motion is kept constant, **(B)** the global tibia position is held constant and **(C)** when the tibia is rotated to match the take-off posture while keeping the rigid levering motion constant.

Contrary to our expectations, when arch recoil was removed from propulsion, talar centroid height was 5.7% ± 2.4 % (*p* < 0.01, *r* = 1) higher and 11.0% ± 3.2 % (*p* < 0.01, *r* = 1) more forward (superior: 70.2 ± 13.3 mm, anterior: 71.8 ± 20.3 mm) than when arch recoil was maintained (superior: 66.5 ± 13.1 mm, anterior: 64.5 ± 17.5 mm) (Figure 3A). Thus, arch recoil appeared to reduce the superior and anterior translation of the talus.

Instead, arch recoil determined the upright posture of the talus. The recoiling medial arch causes the talus to rotate backwards, curling under the tibia during ankle plantarflexion (see Supplementary Video S1). The tibiotalar articular surface of the talus thus faces superiorly at push-off but is globally lower than the modelled rigid arch.

Additionally, to push off with a typical upright tibia posture (Figure 3B), our model shows that the rigid arch would have 29.8% ± 9.0% (0.028 ± 0.008 s) less propulsive ground contact time.

If we allow the same levering motion and tibia lean at push-off with a rigid foot as we observe with a mobile foot, the tibiotalar joint would achieve an extremely plantarflexed articular position with less overlap between joint surfaces (Figure 3C).

**FIGURE 4:**
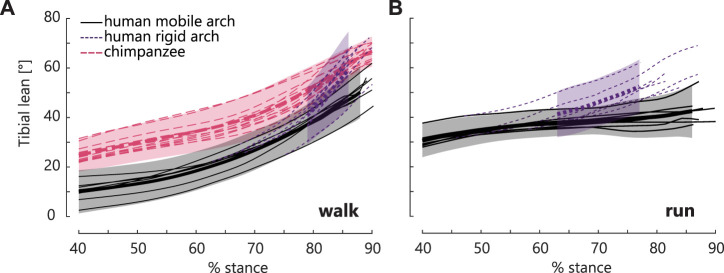
Tibia lean as measured in human and chimpanzee locomotion. **(A)** The measured human mobile arch’s tibia orientation (black, solid line) is compared to the modelled rigid arch’s tibia (purple, dotted line) (*N* = 7, 1 step/participant) and chimpanzee tibia orientation in bipedal walking (pink, dashed line) (*N* = 3, 13 steps). At push-off in walking, the human rigid arch’s tibia is in the same position as the chimpanzee’s. Mean angles are shown in a thicker line, with ± 1.5 SD. **(B)** The human mobile arch (black, solid line) compared to the modelled rigid arch (purple, dotted line) in running.

Arch recoil enabled a more upright tibia. When the arch was locked and the tibiotalar joint motion maintained, the tibia was substantially leaned forward in both walking and running (Figure 4). Further, in walking, the human rigid arch caused tibial lean to approach that of the chimpanzee, whose midfoot plantarflexes minimally after heel lift.

#### 3.2.2 Multiple regression

We used predictor variables of arch recoil range of motion, unloaded static arch angle, and a categorical variable for walking and running. Our multiple linear regression model showed that arch recoil range of motion was a better predictor of the magnitude of anterior and superior displacement of the talar centroid (*R* ^2^ = 0.82, *p* < 0.01, 95%CI: [−5.9,−3.2]) than unloaded static arch angle (*p* = 0.79, 95%CI: [−0.7,0.3]) (Table 1).

**TABLE 1:** Multiple regression parameters for predicting the ankle plantarflexion range of motion and the sagittal plane displacement of the talar centroid.

	Variable	Coefficient (Standard error)	95% confidence intervals	*p*-value
*Ankle plantarflexion range of motion (R* ^*2*^ *= 0.92, adj R* ^*2*^ *= 0.90, F = 40.6, p < 0.01)*	Intercept	−0.58 (4.92)	[−10.22, 9.06]	0.91
	Static arch height	−0.02 (0.09)	[−0.19, 0.14]	0.79
	Arch recoil	0.78 (0.19)	[0.42, 1.14]	< 0.01
	Walk/run (categorical)	−13.38 (1.43)	[−16.18, −10.59]	< 0.01
*Talar centroid displacement (R* ^*2*^ *= 0.82, adj R* ^*2*^ *= 0.77, F = 15.2, p < 0.01)*	Intercept	24.70 (18.12)	[−10.82, 60.21]	0.20
	Static arch height	−0.09 (0.31)	[−0.70, 0.53]	0.79
	Arch recoil	−4.58 (0.68)	[−5.92, −3.24]	< 0.01
	Walk/run (categorical)	−4.56 (5.26)	[−14.87, 5.75]	0.41

Using the same independent variables, we found that arch recoil range of motion better predicted ankle plantarflexion range of motion in propulsion (*R* ^2^ = 0.92, *p* < 0.01, 95% confidence interval on slope (95%CI): [0.4, 1.1]) than unloaded static arch angle (*p* = 0.79, 95%CI [−0.2, 0.1]). Arch and ankle plantarflexion range of motion were also significantly larger in running than in walking.

#### 3.2.3 Contributions of arch joints to talus posture

**FIGURE 5:**
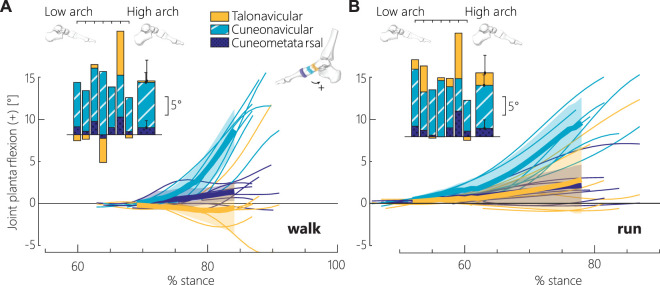
Contribution of the midfoot joints along the first ray to arch recoil (plantarflexion). **(A)** In walking, and **(B)** in running, the joint motion during propulsion is shown relative to peak arch flattening for the talonavicular (gold/light), cuneonavicular (teal/medium), and cuneometatarsal (navy/dark) joints. Each thin line within a color represents one participant (*n* = 7 per joint). The thick lines represent the average with one standard deviation shaded around the mean. The relative contribution of each joint is shown in the inset bar plot, with the plantarflexion angle change between peak arch flattening and push-off (maximum MTPJ dorsiflexion) for each participant. Participants are ordered by unloaded arch plantarflexion angle. The thicker bar represents the mean ± 1 standard deviation. Coordinate systems are aligned with the first metatarsal at peak arch flattening (Supplementary Figure S1).

Here, we consider the medial column of the arch as part of the kinematic chain of the leg. Sagittal medial arch plantarflexion mobility in propulsion is significantly greater in the navicular-medial cuneiform joint (cuneonavicular joint) than both the talonavicular and cuneometatarsal joints (significant effect on joint angle, multiple comparisons: *p* < 0.05) (Figure 5).

## 4 Discussion

### 4.1 COM propulsion

The arch does not seem to recoil to directly propel the center of mass over and above the levering action of the foot. In walking, the timing of COM propulsion does not support a causal relationship between arch recoil and COM motion. In our model, in both walking and running, when the arch was allowed to recoil, the talus was significantly lower and more posterior than when the arch was simulated as rigid. However, *in vivo,* participants with more arch recoil took off from a higher ankle position than those with reduced arch recoil. Thus, this suggests that while arch recoil does not seem to lift the ankle over and above the lever action of the foot (Figure 1A), it enables postural changes that are favorable for propelling the COM. Further research in this area is required to establish causal relationships between arch recoil and COM propulsion.

### 4.2 Posture to support ankle function

The primary finding of this work is that medial column plantarflexion (arch recoil) enables the upright orientation of the talus. Our mathematically rigid arch model suggests that arch recoil does not directly lift the talus over and above the dynamic levering of the foot by the ankle plantarflexors. However, it seems that *in vivo*, participants take advantage of additional arch recoil through reorientation of the talus, which leads to an upright-facing talar articular surface at a higher global ankle position.

Our results imply that humans have an optimal upright tibia orientation for propulsion. When arch recoil was removed from the propulsive motion, the tibia was substantially leaned forward (Figures 3A, 4). If humans were to locomote in this posture, it would require push-off with a flexed knee which would likely reduce the efficiency of human bipedal movement due to muscle compensations at other joints (Waters and Mulroy, 1999). Further, when the take-off position was maintained in the arch bones, and the tibia was then rotated to match the global take-off orientation of the tibia, the tibio-talar joint geometry showed a biologically infeasible posture; in other words, the tibia runs out of plantarflexion room on the talus (Figure 3C). Thus, consistent with our multiple regression model and rigid foot model (Figure 3B), it suggests that with restricted arch recoil, participants most likely take off from a lower position, with reduced MTPJ dorsiflexion and ankle plantarflexion. Analogously, our data show that a recoiling arch enables the talus to be upright, to take off from a higher global position, while ensuring that the tibia can achieve the optimal angle for propulsion.

**FIGURE 6:**
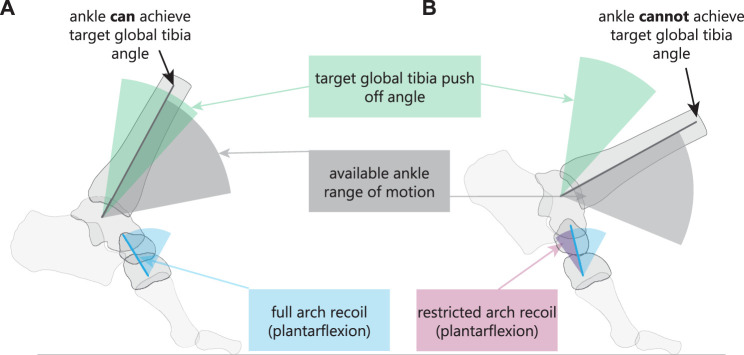
**(A)** Arch plantarflexion at the cuneonavicular joint determines the set of global postures available to the tibia for locomotion. When the arch recoils, the available ankle range of motion falls in the range for optimal tibia push-off posture. **(B)** When arch recoil (plantarflexion) is limited, the ankle range of motion does not enable the tibia to fall in the optimal range for push-off.

We hypothesize that arch recoil supports propulsive ankle function. Though our current analysis is kinematic, several results support this hypothesis. Across participants, increased arch recoil was correlated with ankle plantarflexion range of motion. Thus, as a person’s medial arch is more mobile, the superior talar surface is more upright, increasing the tibia’s available range of motion while remaining relatively vertical (Figure 6). Secondly, our arch model shows that ground contact time would be substantially reduced if the arch did not recoil, because the tibiotalar joint would reach the limits of its posterior point of contact earlier. As a result, we predict that higher muscle forces would be required to maintain the same propulsive impulse. The same ankle plantarflexion would be required for the reduced time period, increasing ankle plantarflexion velocity and requiring higher triceps surae contraction velocities. In general, these results suggest that reduced arch recoil would force the calf muscles to generate propulsive power under unfavorable contractile conditions (Carrier et al., 1994).

Arch and ankle plantarflexion range of motion were significantly larger in running than in walking, suggesting that arch recoil may play a more critical role in maintaining an upright tibia posture in running. The participants in this study maintained a relatively constant tibial posture in late stance during running, supporting the idea that there is an optimal tibial posture for push-off (Figure 4). This is further emphasized by the larger effect of a rigid arch compared to the mobile arch on global tibia posture in running compared to walking (Figure 4). Since the postural advantages of arch recoil are present in walking but play a larger role in running, it is possible that evolutionary pressure for a mobile medial arch was amplified as humans began to run (Bramble and Lieberman, 2004).

### 4.3 Joint contributions

Our results show that medial arch plantarflexion mobility occurs primarily at the cuneonavicular joint in human propulsion, in contrast to the arch mobility of non-human primates which is perceived to occur at the talonavicular, calcaneocuboid and cuboid-metatarsal joints (Elftman and Manter, 1935b; DeSilva, 2009) (Figure 5). The medial column of the arch is a critical part of the kinematic chain of the leg, as arch plantarflexion at the cuneonavicular joint enables ankle plantarflexion within the acceptable range of global tibia push off postures (Figure 6). While the cuneonavicular joint is known to be mobile (Arndt et al., 2007; Lundgren et al., 2008), our results indicate that it is primarily responsible for orienting the superior talar surface upright and enabling locomotion on extended limbs. In contrast, flexed limb walking and a midfoot break is typical in non-human primates. The non-human primate midfoot dorsiflexes in propulsion, instead of plantarflexing as in humans. Based on our results here, we presume that it occurs primarily at the cuneonavicular joint. This is consistent with gorillas and chimpanzees undergoing generally less motion than humans between the navicular and first metatarsal, presumably at the cuneonavicular joint (Negishi et al., 2021). Thus, our divergent evolutionary paths in midfoot function from non-human primates is likely reflected in the morphology and mobility of the cuneonavicular joint. We suggest that future comparative and paleontological analyses should investigate changes in the morphology of the cuneonavicular joint to better understand the evolution of the medial arch.

Our results should be interpreted within several constraints. Firstly, we cannot evaluate arch recoil in the final 10% of stance phase due to the relatively small size of the biplanar videoradiography volume. As a result, we chose to standardize arch recoil from maximal arch flattening to maximal MTPJ dorsiflexion. While this captures most of the push-off phase, there is additional arch recoil that occurs in terminal stance and in early swing phase (Kelly et al., 2014). During this interval, the talonavicular joint plantarflexes more than during early propulsion (Arndt et al., 2007; Lundgren et al., 2008), suggesting there may be a distal to proximal sequence to arch recoil. Further, to our knowledge, these are the first non-invasive measurements of *in vivo* midfoot joint motion using biplanar videoradiography. Our understanding of foot joint motion has been bolstered by measurements using bone pins, however, this invasive technique may have affected normal walking kinematics. Finally, our choice to align all coordinate systems with the first metatarsal’s at the point of maximum arch flattening may lead to angle cross-talk relative to published midfoot joint angles. Other authors have defined coordinate systems to reflect individual bone morphology, or changes relative to a standing posture, which will inevitably change the magnitude of angles in each plane. Here, the advantage to using the aligned coordinate systems at a specific gait event is that they enable the standardized measurement of joint contributions across participants.

### 4.4 Evolutionary implication for the evolution of upright bipedalism

**FIGURE 7:**
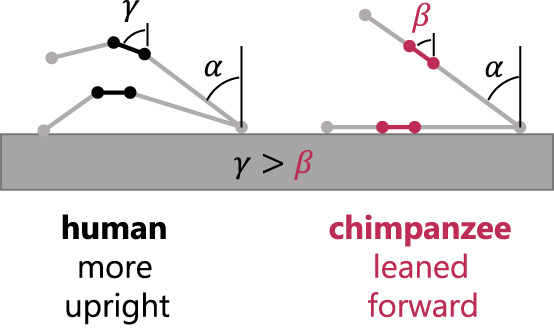
The talus/midfoot line segment (black for the human rigid arch, pink for chimpanzee) at the same global metatarsal position in the levering process, without arch recoil. The talus/midfoot segment is naturally more superiorly oriented in the human arch due to its raised posture. Human recoil further orients the talus upright, while the midfoot break in chimpanzees would further lean the talus forward.

Neither ankle plantarflexion nor global talar position at push-off were related to static arch height, despite being strongly correlated to arch plantarflexion mobility. This suggests that our ability to locomote bipedally, specifically during push-off, is more strongly related to the plantarflexion mobility of our arch than its posture, in contrast to the many evolutionary studies that infer the origins of bipedalism primarily from the presence of a raised medial arch. We hypothesize, based on our results here, that arch plantarflexion mobility is a fundamental piece of the evolutionary puzzle. Specifically, there may be a functional link between arch structure and arch recoil in propulsion that may help humans move bipedally compared to other primates. Humans locomote over an extended lower limb, thus requiring an upright tibia orientation compared to chimpanzees’ flexed limb walking posture (Figure 4A). We propose that humans may have evolved a pronounced medial arch for two non-mutually exclusive reasons. First, the arch naturally orients the talus’ superior articular surface upright, such that even without arch recoil, it is more upright than in the non-arched feet of chimpanzees (Figure 7). Secondly, the arch-spanning tissues in humans have a longer moment arm about the midfoot joints to produce more arch recoil than in chimpanzees. Thus, in addition to the natural upright orientation of the human talar superior surface, arch recoil further enables propulsion over a longer period while the tibia can remain upright. When chimpanzees experience a midfoot break, the talus and tarsal bones lean forward, which, in concert with anatomical limitations at the knee and hip, likely contributes to chimpanzees’ flexed-limbed posture in bipedal push-off. Due to their lack of arch, the midfoot has less capacity to recoil and re-orient the talus to be upright. These ideas are consistent with reduced midfoot recoil and reduced ankle push-off power in chimpanzees compared to humans (Holowka et al., 2017a; O’Neill et al., 2022). Overall, we hypothesize that a prerequisite for hominins to push off efficiently with a fully extended leg was the evolution of a structural arch to function in tandem with the recoiling arch. These ideas should be investigated in future studies.

### 4.5 Applications

Enabling arch plantarflexion mobility has many important applications, including footwear design, understanding pathology, and surgical practice. Certain footwear modifications, such as increasing the bending stiffness of the shoe’s sole, or wearing arch-restricting inserts, both reduce arch plantarflexion and can respectively modify the ankle’s muscular contractile conditions during locomotion (Cigoja et al., 2020), or increase the metabolic cost of level ground running (Stearne et al., 2016). Our results also have implications for people with naturally stiff feet or foot pathologies (such as osteoarthritis) that reduce mobility in the arch. When the tarsal joints are surgically fused, ankle power is decreased in walking, further suggesting that a mobile arch supports ankle propulsion (Beischer et al., 1999). Our method could also be used to predict dynamic motion patterns in surgical joint fusions. By mathematically fixing joints in known positions, we can elucidate potential changes along the kinematic chain. For example, we would expect that fusing the cuneonavicular joint would substantially impact propulsion, causing the foot to leave the ground early or increasing force requirements at the ankle. These results highlight the importance of preserving arch mobility in surgical practice and footwear design.

In conclusion, in bipedal walking and running, human medial arch recoil works in tandem with the morphology of the medial arch to facilitate upright locomotion through its effect on talus posture, ankle range of motion and ground contact time. We argue that while differences in medial arch height may visually distinguish hominins from other primates, our arch plantarflexion mobility is more critical to our ability to locomote on two feet. Thus, mapping morphology-mobility relationships in our extant relatives and humans, as well as forward-dynamic predictions of the fossil record, are necessary to understand our ancestors’ locomotory patterns.

## Data Availability

The dataset and code to produce the results in this manuscript are stored in a Dryad repository here: https://doi.org/10.5061/dryad.866t1g1vt. Any additional code or data supporting the conclusions in this article are available upon reasonable request to the corresponding author.
