# Supplementary material for: Mobility of the human foot’s medial arch helps enable upright bipedal locomotion
Source: Front Bioeng Biotechnol. 2023 May 30;11:1155439. doi: 10.3389/fbioe.2023.1155439 (PMC10264861; doi:10.3389/fbioe.2023.1155439)
Supplement: Supplementary file 2 [file DataSheet1.docx]

Supplementary Material

Mobility of the human foot’s medial arch helps enable upright bipedal locomotion

Lauren Welte^1^*†; Nicholas B Holowka^2^; Luke A Kelly^3^; Anton Arndt^4, 5^; Michael J Rainbow^1*^

^1^ Department of Mechanical & Materials Engineering, Queen’s University, Kingston, ON, Canada

2 Department of Anthropology, University at Buffalo, Buffalo, NY 14260, USA

3 School of Human Movement and Nutrition Sciences, University of Queensland, Brisbane, QLD 4072, Australia

4 The Swedish School of Sport and Health Sciences (GIH), Stockholm, Sweden

5 Karolinska Institute, Stockholm, Sweden

† Current address: Department of Mechanical Engineering, University of Wisconsin-Madison, Madison, WI, USA

*** Correspondence:**Lauren Welte or Michael J Rainbow
[l.welte@queensu.ca](mailto:l.welte@queensu.ca) or [michael.rainbow@queensu.ca](mailto:michael.rainbow@queensu.ca)

# Supplementary Video

**Movie S1 (separate fil**e). Motion of the recoiling and modelled rigid medial arch. The levering motion of the first metatarsal is included on the right, but has been removed to show the relative motion of the recoiling arch to the rigid arch on the left.

# Supplementary Figure


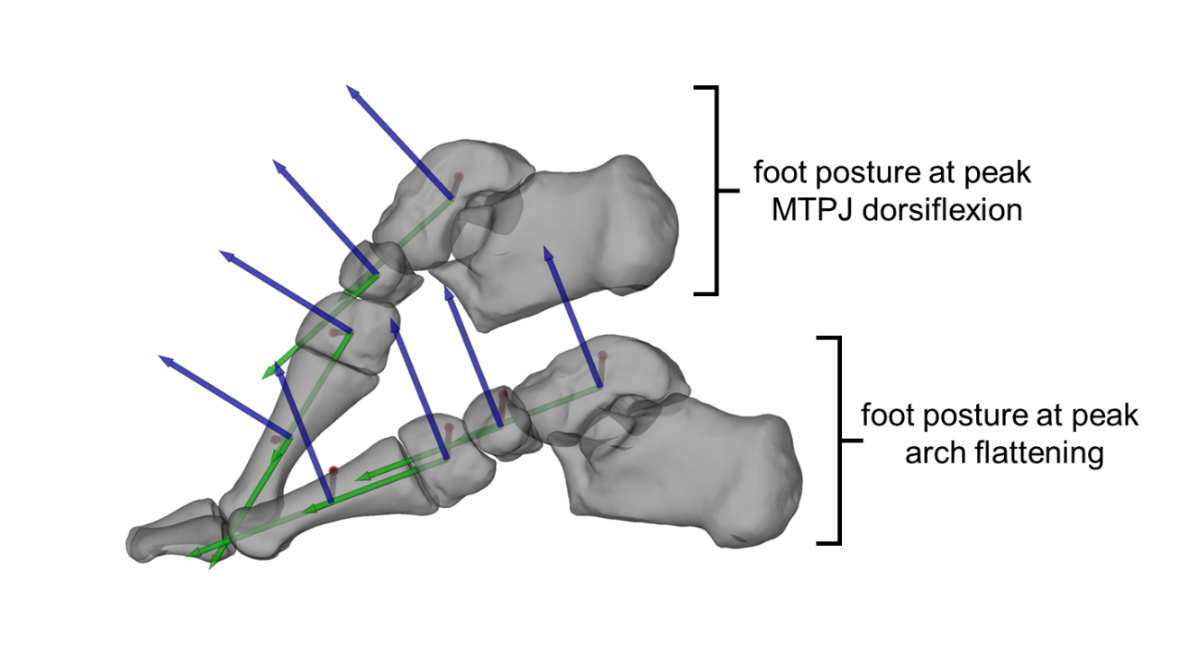


**Fig. S1**. Medial arch co-ordinate systems. Co-ordinate systems of the arch bones, fixed to align with the orientation of the inertial co-ordinate system of the first metatarsal at peak arch flattening. A representative participant’s foot posture is shown during a run at peak arch flattening and peak metatarsophalangeal joint (MTPJ) dorsiflexion.

# Supplementary Methods

Here, we provide the math that rigidly locks the arch, and the cases demonstrated in *Mobility of the human foot’s medial arch helps enable upright bipedal locomotion.*

## Conventions:

[T] is the 4x4 matrix that moves a rigid body from computed tomography (CT) space to x-ray (xr) space. T is composed of a 3x3 rotation matrix and 3x1 translation matrix.

| $\left[ T_{xr\leftarrow CT} \right]=\left[ \begin{matrix} \left[ R \right] & \left[ t \right] \\ \begin{matrix} 0 & 0 & 0 \end{matrix} & 1 \end{matrix} \right]$ | Eq. 1 |
| --- | --- |

The points on a bone can be moved using Eq. 1:

| $\left[ \begin{matrix} p_{x_{xr}} \\ p_{y_{xr}} \\ {p_{z}}_{xr} \\ 1 \end{matrix} \right]=\left[ T_{xr\leftarrow CT} \right]\left[ \begin{matrix} p_{x_{CT}} \\ p_{y_{CT}} \\ {p_{z}}_{CT} \\ 1 \end{matrix} \right]$ | Eq. 2 |
| --- | --- |

The transform for a specific bone (i.e. mt1 = first metatarsal) is denoted in superscript of the transform. The frame (i) is given outside the brackets (peak arch flattening = pa).

| $\left[ T_{xr\leftarrow CT}^{mt1} \right]_{i=pa}$ | Eq. 3 |
| --- | --- |

All output transforms given here transform the bone’s vertices in CT space to x-ray space.

## Case 1: Fix the arch bones rigidly with the first metatarsal at peak arch flattening

Lock the arch

To fix the talus (tal) at any frame (i) with the first metatarsal (mt1) at peak arch flattening (i=pa):

| $\left[ T_{xr\leftarrow CT}^{tal/mt1} \right]_{i}=\left[ T_{xr\leftarrow CT}^{mt1} \right]_{i}\left[ T_{xr\leftarrow CT}^{mt1} \right]_{i=pa}^{-1}\left[ T_{xr\leftarrow CT}^{\mathrm{tal}} \right]_{i=pa}$ | Eq. 4 |
| --- | --- |

This locks the position of the talus relative to the first metatarsal at the frame pa, and then moves it with the first metatarsal’s motion.

## Case 2: Maintain some joints’ natural motion, but still lock the arch with the first metatarsal

Lock the arch, but maintain tibio-talar motion

The arch is locked to the first metatarsal below the tibiotalar joint. To maintain the relative motion of the tibia (tib) relative to a talus fixed with the first metatarsal:

| $\left[ T_{xr\leftarrow CT}^{tib/tal/mt1} \right]_{i}=\left[ T_{xr\leftarrow CT}^{mt1} \right]_{i}\left[ T_{xr\leftarrow CT}^{mt1} \right]_{i=pa}^{-1}\left[ T_{xr\leftarrow CT}^{\mathrm{tal}} \right]_{i=pa}\left[ T_{xr\leftarrow CT}^{\mathrm{tal}} \right]_{i}^{-1}\left[ T_{xr\leftarrow CT}^{\mathrm{tib}} \right]_{i}$ | Eq. 5 |
| --- | --- |

Note that the first three transforms can simplify to Eq. 4.

## Case 3: Fix a joint to the position at a different time than when the arch is locked

Lock the arch at peak arch flattening, but keep the tibio-talar joint in the take-off (to) position.

| $\left[ T_{xr\leftarrow CT}^{tib/tal/mt1} \right]_{i}=\left[ T_{xr\leftarrow CT}^{mt1} \right]_{i}\left[ T_{xr\leftarrow CT}^{mt1} \right]_{i=pa}^{-1}\left[ T_{xr\leftarrow CT}^{\mathrm{tal}} \right]_{i=pa}\left[ T_{xr\leftarrow CT}^{\mathrm{tal}} \right]_{i=to}^{-1}\left[ T_{xr\leftarrow CT}^{\mathrm{tib}} \right]_{i=to}$ | Eq. 6 |
| --- | --- |

Again, note the first three transforms are the same as Eq. 4. The only difference between Eq 5 and 6 are the frame at which the last two transforms are taken.
